# Predictors of Health-Related Quality of Life in Older Adults After a Flood

**DOI:** 10.1093/geroni/igaf122.1975

**Published:** 2025-12-31

**Authors:** Katie Cherry, Piper Bordes, Luke Miller, Matthew Calamia, Emily Elliott

**Affiliations:** Louisiana State University, Baton Rouge, Louisiana, United States; Louisiana State University, Baton Rouge, Louisiana, United States; Louisiana State University, Baton Rouge, Louisiana, United States; Louisiana State University, Baton Rouge, Louisiana, United States; Louisiana State University, Baton Rouge, Louisiana, United States

## Abstract

Multiple severe weather events may threaten health and well-being for those who live in disaster-impacted communities. In 2005, coastal residents of south Louisiana experienced catastrophic damage in Hurricane Katrina which forced many to permanently relocate inland to higher and presumably safer ground. In August of 2016, historic flooding brought widespread destruction across a 22-parish (county) area, creating additional loss for those who had moved to Baton Rouge, Louisiana after Katrina. This study is part of a larger longitudinal research project on health indicators after multiple disasters. Here we examined health-related quality of life assessed by the SF-36 Health Survey (Ware & Sherbourne, 1992). Three flood exposure groups were compared across two waves of testing: non-flooded (controls), single disaster (flooded in 2016) and double disaster (flooded in 2005 and again in 2016). The flood exposure groups did not differ in physical health. Mental health was poorer for those whose homes flooded compared to the non-flooded controls, although their scores improved somewhat by the second wave of testing. Regression analyses confirmed that age, prior lifetime trauma, and intolerance of uncertainty (IUS) at Wave 1 predicted physical health at Wave 2, although IUS lost its significance when agency was added as a predictor. In contrast, age and IUS predicted mental health and these variables held their significance when agency was accounted for. These data show that persons who experience difficulty tolerating ambiguous or uncertain situations may be at greater risk for post-disaster distress when navigating the challenges of long-term flood recovery.

